# Intraneural Masson Tumor of the Hand

**Published:** 2019-02-26

**Authors:** Alexander Lauder, Rex C. Bentley, Suhail K. Mithani

**Affiliations:** Duke University Medical Center, Durham, NC

**Keywords:** Masson tumor, intravascular papillary endothelial hyperplasia, IPEH, hand mass, intraneural vascular tumor

## DESCRIPTION

A 19-year-old man presented with a mass of the volar third web space after blunt trauma. It was 3 × 2 cm, tender, noncompressible, nonpulsatile, and without associated neurovascular deficits. Surgical exploration revealed the mass to be within a common digital nerve. Pathology was consistent with a Masson tumor.

## QUESTIONS

1. What is a Masson tumor?
2. What is the typical presentation?
3. How are these tumors diagnosed?
4. What makes this case unique and what is the typical treatment?

## DISCUSSION

A Masson tumor, or intravascular papillary endothelial hyperplasia (IPEH), is an exaggerated reparative phenomenon representing recanalization and organization of a thrombus within a normal vessel, vascular malformation, or hematoma. It is a rare tumor accounting for approximately 2% to 4% of vascular tumors of the skin and subcutaneous tissue.^1^ The diagnosis was first described by Pierre Masson in 1923.

**Figure 1:**
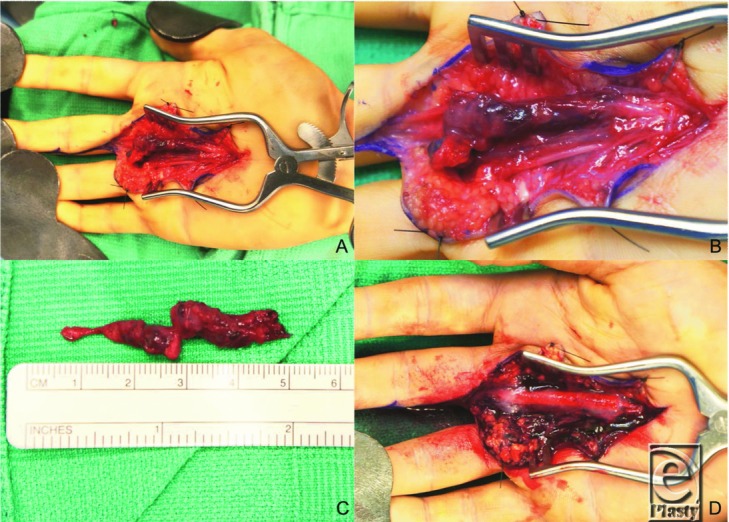
Intraoperative photographs showing the Masson tumor in situ (*a*) magnified (*b*), after surgical excision (*c*), and after nerve protector and fibrin glue application (*d*).

**Figure 2:**
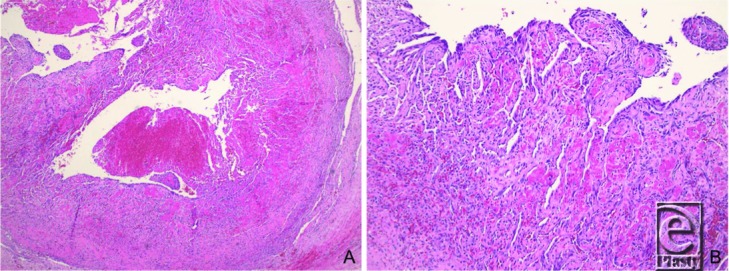
Low- (*a*) and high-power (*b*) micrographs identifying intravascular organized thrombi and areas of florid endothelial proliferation forming secondary lumina and papillary structures.

Presentation is typically a well-defined, compressible, small (<5 cm), slow-growing, nonpulsatile mass that may be either painful or painless. IPEH can occur in any location throughout the body but is most commonly found in the head and neck and extremities. It is typically found in the skin and subcutaneous tissue and is usually associated with a blood vessel. In this particular case, the mass was found to be adherent to the common digital nerve of the ring and long fingers (Figs 1*a* and 1*b*) and intrafascicular dissection with sacrifice of roughly 10% of the fascicles was required to resect the mass (Fig 1*c*). The common digital artery was found to be nonadherent to the mass and was easily dissected free distally to normal appearing digital arteries. The significant intrafascicular adherent dissecting planes along the course of the mass suggested a perineural vascular origin. The residual nerve was wrapped with a nerve protector (AxoGuard, AxoGen Inc, Alachua, Florida) and sealed with fibrin glue to facilitate nerve regeneration (Fig 1*d*). Pathology identified the mass as IPEH or Masson tumor (Fig 2).

A Masson tumor is diagnosed histologically by inflammatory reactive hyperplasia of endothelial cells lined with small papillary structures and hyaline stalks with organized thrombus, minimal nuclear atypia, and absence of necrosis (Fig 2).^2^ Histopathology can mimic that of malignant angiosarcoma, indicating the importance of accurate diagnosis. Three types of IPEH have been described: (1) primary or pure (33%-56%), in which the lesion arises de novo from a dilated vessel (typically a vein); (2) secondary or mixed (40%-60%), in which the mass develops in relation to a preexisting vascular malformation such as hemangioma, lymphangioma, pyogenic granuloma, or arteriovenous fistula; and (3) extravascular (4%-7%), arising from a reactive process related to hematoma.

To our knowledge, this is the first reported case of an intraneural IPEH requiring intrafascicular dissection of a digital nerve. Other reports describe close proximity to the underlying neurovascular structures without involvement of neurovascular tissue planes.^3^ The mass involvement with the underlying nerve was not suggested on initial physical examination, as this patient presented without Tinel sign or neurological deficit. The extensive infiltration of the common digital nerve found on surgical exploration suggests mass originated from either (1) the perineural vasculature of the common digital nerve or (2) an underlying vascular malformation (secondary IPEH).^4^ Complete surgical resection is therapeutic but can be associated with complication. At initial postoperative follow-up, the patient endorsed decreased sensation along the ulnar border of the long finger, which resolved after 8 months. This case highlights the difficulty in clinically differentiating between apparently benign lesions that are amenable to routine excision and those with more complex involvement with underlying structures.
